# Perceived barriers to early mobilization of intensive care unit patients by nurses in hospitals affiliated to Jundishapur University of Medical Sciences of Ahvaz in 2019

**DOI:** 10.25122/jml-2019-0135

**Published:** 2021

**Authors:** Mahbubeh Babazadeh, Simin Jahani, Tayebeh Poursangbor, Bahaman Cheraghian

**Affiliations:** 1. Nursing Care Research Center in Chronic Diseases, School of Nursing and Midwifery, Ahvaz Jundishapur University of Medical Sciences, Ahvaz, Iran; 2. Student Research Committee, Ahvaz Jundishapur University of Medical Sciences, Ahvaz, Iran; 3. Department of Biostatistics and Epidemiology, School of Public Health, Ahvaz Jundishapur University of Medical Sciences, Ahvaz, Iran

**Keywords:** early mobilization, intensive care units, nurse, barrier to early mobilization

## Abstract

Early mobilization (EM) of patients in the intensive care unit (ICU) is a safe, feasible, and beneficial approach. However, the implementation of EM as a part of routine clinical care can be challenging. As a result, the present study aimed to identify the potential barriers to EM of ICU patients. The statistical population of this descriptive-analytical study included 107 critical care nurses working in hospitals affiliated with the Jundishapur University of Medical Sciences of Ahvaz. The participants were selected using the census method among the eligible critical care nurses, and the researcher-made questionnaire was used for data collection. This questionnaire included a demographic questionnaire and an inventory of barriers to EM. In total, 72% of the nurses had a highly positive attitude towards EM implementation, whereas relatively few had a slightly positive attitude. The major human-resource-related barriers included the lack of trained staff (76.6%), inadequate shift nurses (74%), and inadequate time for this procedure (57.9%). Approximately 88.9%, 82.2%, 62%, and 57.9% of the nurses reported coma or a deep degree of sedation, mobilization of obese patients, mobilization of patients with agitation, and pain, respectively, as the major patient-related barriers. The lack of EM implementation and recording according to the checklist (90.4%), the lack of an approved EM implementation protocol (88.8%), and inadequate equipment for the mobilization of mechanically ventilated patients (58%) were among the major equipment-related barriers. The participating nurses were aware of the EM advantages, and the majority of them had a highly positive attitude towards its implementation in the ICU. However, nurses believed that the actual EM implementation is associated with challenges such as human resources limitations, equipment-related barriers, and patient-related barriers.

## Introduction

In many intensive care units (ICUs), routine procedures, including a deep degree of sedation and bed rest, are carried out to manage critically ill patients [1]. As a result, patients in the ICU, especially mechanically ventilated patients, experience a lower degree of mobilization. This reduction can cause weak mobilization performance [2, 3]. This immobilization can reduce muscle strength, increase mechanical ventilation duration, and prolong the length of hospital stay [4]. Moreover, the functional disorders following ICU hospitalization reduce the quality of life of the ICU patients, thereby increasing the need for long-term nursing care [2]. There are some potential barriers to EM in ICU patients, such as the low ratio of nursing and physiotherapy staff to patients, arterial lines and tubes, deep degree of sedation, informed refusal, and unit culture [1, 5]. Critical care nurses should play a crucial role in shifting towards improved care quality owing to their understanding of patients’ health conditions and their needs within a local context. Meanwhile, nurses perceived patient safety concerns, lack of education, or trained workforce as the significant barriers for EM more than other healthcare providers. Such a difference in recognition can lead to a gap in mobilization performance among other healthcare professionals [6].

EM can be defined as “the application of physical activity within the first two to five days of critical illness or injury,” which continues during the ICU stay by the healthcare providers [3, 7]. EM is a complex intervention that requires accurate assessment and management of the patients and also training and collaboration of the interdisciplinary teams [8]. Results from different studies have shown that this initial intervention in ICU is a highly potential procedure. The reported advantages in different studies include the reduced length of ICU stay, improved quality of life, and mentality, respiratory conditions, and improved functional strength of the patients [7–11]. Despite these potential benefits, the provision of EM has not been widely acknowledged [2]. A recent 1-day point-prevalence study in Germany showed that only 24% of all mechanically ventilated patients and only 8% of patients with an endotracheal tube were mobilized out of bed as a part of routine care. Moreover, a similar 1-day study in Australia and New Zealand showed that none of 224 patients requiring mechanical ventilation stood or were ambulated over the study day [12].

Quantitative studies were conducted to understand the barriers to EM of ICU patients. Some studies on healthcare quality improvement intended to understand whether the clinicians’ attitude toward EM is a barrier to its delivery [13, 14]. These studies showed that individual and patient safety and the lack of clinician understanding are potential barriers to EM of ICU patients [2, 14]. Moreover, results from a recent study showed that nurse-related barriers to EM were the lack of training, discomfort, and inadequate time to mobilize the patients. The major barrier reported by the nurses was “heavy workload” [15]. Nurses, working to care for patients and support their colleagues, have an essential role in applying EM practices for ICU patients.

Moreover, nurses spend more time with patients than do any other healthcare providers. As a result, they are important and key members and a linkage between the patient and other medical staff. They also have a great role in training medical staff and patients and better EM implementation [16].

Despite the important role of nurses in EM of ICU patients, there is no study on identifying the nurses’ perception of barriers to EM in Iran. Therefore, the present study aimed to assess the attitude and awareness of nurses towards EM and to understand potential barriers to EM in the ICU and critical care unit (CCU) patients in teaching hospitals of Ahvaz.

## Material and Methods

The statistical population included 107 critical care nurses in the hospitals affiliated with the Jundishapur University of Medical Sciences of Ahvaz, Iran (Imam Khomeini Hospital, Golestan Hospital, Razi Hospital, Sina Hospital, Taleghani Hospital, and Shahid Baghaei Hospital 2). In these hospitals, there is an average of 12 beds in each ICU ward. The nurse-to-patient ratio is 1:4. In the hospitals that were examined in this study, the prohibition of visits to patients admitted to ICUs is applied. The sampling was done in a period spanning 21 March and 23 August 2019. Subjects were selected using the census method. In that, the eligible critical care nurses were selected and enrolled. The inclusion criteria included having at least a master’s degree in nursing and 1-year work experience in the ICU. The exclusion criterion was returning an incomplete questionnaire. During the research period, the informed written consent of 196 nurses was collected. However, only 107 nurses completed their questionnaires.

It was a voluntary survey, and only data of participants who gave consent were used in the study. 

Data collection instruments included a researcher-made questionnaire with two parts, namely demographic information and barriers to EM implementation. The demographic questionnaire included data on age, sex, educational attainment, work experience in the ICU, and type of ICU. Also, the inventory of barriers to EM implementation had two sections. The first section was human-related barriers and the second section was equipment-related barriers. The human-related barriers were comprised of 4 parts, namely limitations of human resources, level of knowledge, the attitude of nurses, and patient-related barriers. The inventory of attitude barriers was based on a 5-point Likert scale from completely agree to completely disagree. The minimum and maximum scores of this 9-item inventory of attitude barriers were 9 and 45, respectively. Positive items are scored from “1- completely disagree” to “5- completely agree”, whereas the negative items are scored from “1 – completely agree” to “5- completely disagree.” With respect to the attitude of the nurses towards EM, scores ≤26, 27–35, and ≥36 represented slightly, moderately, and highly positive, respectively. The assessment of knowledge-related barriers had 11 items, which scored 1 for each correct response and 0 for each incorrect response. Therefore, the highest and lowest scores were 11 and 0, respectively. Concerning the knowledge, scores ≤5, 6–8, and ≥9 represented low, moderate, and high knowledge of the nurses of the benefits of EM, respectively. The five items on limitations of human resources are scored with 1 for “agree” and 0 for “disagree” responses. Therefore, the lowest and highest scores in this section are 0 and 5, respectively. The patient-related barriers section has six items. Items in this section are scored similarly to the limitations of human resources, with the lowest and highest scores of 0 and 6, respectively. The equipment-related barriers section has three items, which are scored with 1 for “agree” and 0 for “disagree” responses. Therefore, the lowest and highest scores are 0 and 3, respectively. In the present study, Cronbach’s alpha of the inventory of barriers to EM for the limitations of human resources, level of knowledge, the attitude of nurses, and patient-related barriers were 0.79, 0.88, 0.87, 0.89, and 0.91, respectively. Cronbach’s alpha of equipment-related barriers was 77.

Data analysis was done using the SPSS software, version 21. The descriptive statistics, such as tables of frequency and percentage, were used to present human- and equipment-related barriers.

## Results

Among the participants, 67 nurses (62.6%) were women, and 40 nurses (37.4%) were men. The majority of them were in the age range of 26–36 years old. According to data, 101 (94.4%) of the nurses had a bachelor’s degree in nursing, and about 6 (5.6%) had a master’s degree in nursing. Moreover, 43 (40.2%) and 64 (59.8%) worked in the CCUs and ICUs, respectively. The majority of the participants had work experience of more than five years, and about 10 (9.3%) had work experience of fewer than five years in intensive care units.

Table 1 presents the results from attitude- and knowledge-related barriers. Results from knowledge-related barriers showed that about 63 (58.9%), 26 (24.3%), and 18 (16.8%) of the participants had high, medium, and low levels of knowledge, respectively. Moreover, results from the assessment of attitude barriers showed that 77 (72%) and about 26 (24.3%) of the participants had a highly positive and moderately positive attitude, respectively. A relatively low number of participants – 4 (3.7%) had a slightly positive attitude.

**Table 1.** Level of knowledge and attitude of nurses towards EM implementation for ICU patients.

**Barriers**	**Low n (%)**	**Medium n (%)**	**High n (%)**
**Knowledge**	18 (16/8)	26 (24/3)	63 (58/9)
**Attitudes**	4 (3/7)	26 (24/3)	77 (72)

Results from the assessment of limitations of human resource showed that the lack of trained staff to implement EM (76.6%), inadequate shift-work nursing staff (74%), and inadequate time for EM implementation (57.9%) were the major barriers to EM in ICU patients (Table 2).

**Table 2.** Frequency and percentage of limitations of human resources frequency as a barrier to EM of ICU patients from views of critical care nurses.

Item	n (% agree)
Regarding the lack of adequate shift nurses, the patients’ companions were responsible to mobilize the patients.	36 (33/6)
Despite the nursing staff shortage, changing the position of the patients is upon the shift nurses.	22 (20/6)
Changing the patient's position requires skilled staff with a predefined job description	82 (76/6)
I do not have adequate time to spend on this procedure.	62 (57/9)
There is not adequate personnel in each shift to perform this procedure.	78 (74)

To critical care nurses, the major patient-related barriers were coma or deep degree for sedation (88.9%), mobilization of obese patients (82.2%), mobilization of patients with agitation (65%), and EM-induced pain in mechanically ventilated patients (57.9%) (Table 3).

**Table 3.** Frequency and percentage of patients-related barriers to EM implementation frequency for ICU patients from views of critical care nurses.

**Item**	**n (% agree)**
It is difficult to mobilize obese patients.	88 (82/2)
Coma or deep degree of sedation inhibits patient mobilization.	95 (88/9)
The mechanically ventilated patients and their families fear mobilization and refuse it.	62 (57/9)
It is a painful procedure for mechanically ventilated patients.	69 (65)
Mobilizing ICU patients may cause respiratory distress or hemodynamic instability.	39 (36/4)
It is difficult to mobilize patients with agitation.	48 (44/8)

Based on the results, the lack of EM implementation and recording based on the checklist (90.7%), the lack of an approved protocol for EM implementation (88.8%), and inadequate equipment for mobilization of mechanically ventilated patients (58%) were the major equipment-related barriers (Table 4).

**Table 4.** Equipment-related barriers to EM of ICU patients from the view of critical care nurses.

Instrument item	**n (% disagree)**
In my unit, there is adequate equipment to mobilize mechanically ventilated patients.	62 (58)
In my unit, there is an approved EM protocol.	97 (90/7)
In my unit, EM is implemented and recorded according to the checklist.	95 (88/8)

## Discussion

Although EM benefits for ICU patients have been well documented in recent years, many intensive care units are not able to effectively implement and integrate EM in daily exercises of the patients [17]. Therefore, the present study aimed to assess the attitude and awareness of nurses towards the benefits of EM and to understand potential barriers to EM in ICU patients hospitalized in teaching hospitals of Ahvaz. Results from the assessment of the attitude and knowledge-related barriers (Table 1) showed that the majority of critical care nurses were aware of the EM benefits, such as reduced length of ICU and hospital stay, improved respiratory system, improved psychological-mental conditions, improved musculoskeletal system, and reduced duration of mechanical ventilation. Moreover, the majority of the participants acknowledged the high importance and priority of EM and emphasized its benefit over its risk. Phua *et al.* proposed that the barriers in clinical guideline implementation can be categorized into knowledge-, attitude-, and behavior-related barriers [18]. Regarding that the participants had a high level of knowledge about the benefits and advantages of EM, it seems that the knowledge and attitude of the nurses cannot be regarded as a barrier to EM in ICU patients. The results of this study were consistent with the recent studies into the knowledge and attitude of the multitask medical team [2, 14]. 

Different studies have shown that compared to rehabilitation therapists, nurses reported the lack of trained staff as a major barrier to EM of the patient [15]. Consistent with these findings, the participants of the current study reported the lack of trained staff in EM implementation in ICU patients (Table 2) as a major barrier related to human resource limitation. This is not surprising since rehabilitation therapists are specifically trained in EM as an essential component of their daily clinical work [15, 19]. Therefore, providing nurses with adequate EM training may be an important educational component for a successful health-promoting project for ICU and CCU patients.

However, increasing patient mobilization in ICU and CCU requires adequate time and nurse staffing [15]. Our participants reported inadequate numbers of nurses and the amount of time to spend on this procedure as major barriers to EM (Table 2). Zhu *et al.* showed that critical care nurses have more responsibilities for mobilizing patients compared to other healthcare providers; therefore, inadequate resources, including numbers of nurses, affect EM implementation in intensive care units [20]. Consistent with these findings, Hopkins *et al.* reported an increased ratio of patients to nurses and the lack of time as major barriers to EM of ICU patients [21].

Patient safety is essential in any exercise intervention. Patients with critical conditions, coma, and a deep degree of sedation are highly vulnerable. Therefore, maximum care should be provided to protect patient safety [22]. The results showed that most nurses believe that a coma or deep degree of sedation is a major barrier to EM implementation (Table 3). Among other barriers to EM implementation in this study were obesity and painful EM procedures in mechanically ventilated patients (Table 3). The results from other studies indicated obesity, mechanical ventilation, endotracheal tube, and unwanted extraction of such tubes as catheters as the major patient-related barriers to EM implementation [23, 24], which are consistent with the findings of the current study. Therefore, concerns regarding patient’s weight and mobilization of mechanically ventilated patients necessitate education and guidance about patient management techniques and a better understanding of probable benefits of mobilization equipment [23]. 

Typically, EM is initiated according to a certain protocol and after cardiorespiratory and neuromuscular stabilization. Many protocols have been separately created [7]. Nevertheless, intensive care units in Iran do not apply any EM implementation protocol. As a result, the lack of a certain protocol is a challenge facing nurses in assessing the patient and EM initiation (Table 4). The lack of EM implementation and recording based on simple checklists can diminish the effectiveness of treatment and nursing care in intensive care units [2]. In the present study, most participants reported the lack of a certain checklist for EM implementation and recording as a barrier to EM implementation.

However, this study has several limitations. For example, participants of this study only represented a narrow spectrum of healthcare providers. This study revealed only some of the potential barriers to EM of patients in intensive care units. Therefore, a larger-scale survey is required. Moreover, the present study did not address the attitude of managers and decision-makers, and it seems that understanding their attitudes towards barriers to EM implementation at the hospital level is essential for resource assignment and enrollment of trained staff in future studies. Also, the sample shows a small sample of health care providers in a city in Iran. Also, the data from this study are only from a single country and only show the opinion of nurses working in the intensive care unit, while other opinions of health care providers, including doctors, may not be relevant. Therefore, future research is needed to better understand the barriers to early mobility that include the views of other health care providers.

## Conclusion

In general, the majority of critical care nurses in teaching hospitals of Ahvaz are aware of the potential benefits of EM implementation for patients with critical conditions and have a highly positive attitude towards it. However, they mentioned many barriers caused by human resources limitations, patient-related barriers, and equipment-related barriers. The results revealed many barriers to EM of ICU patients from the views of critical care nurses. As a result, the findings of this study can be used to train these clinicians and also to develop and implement organizational protocols.

## Acknowledgments

### Funding

Hereby, all participants and the Jundishapur University of Medical Sciences of Ahvaz, which financially supported this study, are thanked. This paper is extracted from the NCRCCD-9736 project approved by Jundishapur University of Medical Sciences of Ahvaz under the code IR.AJUMS.REC.1397.891.

### Ethical approval

The approval for this study was obtained from the Ethics Committee of Ahwaz Jundishapur University of Medical Sciences (approval ID: NCRCCD-9736).

### Consent to participate

Written consent was obtained from each participant.

### Conflict of interest

The authors declare that there is no conflict of interest.
